# Fenestrations of cerebral arteries and their correlation with brain aneurysms

**DOI:** 10.3389/fnana.2025.1523305

**Published:** 2025-03-26

**Authors:** Mila Ćetković, Jelena Boljanović, Ema Bexheti, Filip Vitošević, Damljan Bogićević, Sonja Milašinović, Sadi Bexheti, Dejan Ćetković, Aleksandra Dožić, Milan Milisavljević

**Affiliations:** ^1^Faculty of Medicine, Institute of Histology and Embryology, University of Belgrade, Belgrade, Serbia; ^2^Laboratory for Vascular Morphology, Faculty of Medicine, Institute of Anatomy, University of Belgrade, Belgrade, Serbia; ^3^Faculty of Medical Science, Institute of Anatomy, State University of Tetova, Tetova, North Macedonia; ^4^Special Hospital for Cerebrovascular Diseases “Sent Sava,” Faculty of Medicine, University of Belgrade, Belgrade, Serbia; ^5^Special Hospital for Cerebrovascular Diseases “Sent Sava,” Faculty of Medical Sciences, University of Kragujevac, Kragujevac, Serbia; ^6^Clinical Centre of Montenegro, Institute for Children’s Disease, Podgorica, Montenegro; ^7^Faculty of Dental Medicine, Institute of Anatomy, University of Belgrade, Belgrade, Serbia; ^8^Academy of Medical Sciences, Serbian Medical Association, Belgrade, Serbia

**Keywords:** fenestrations, aneurysms, cerebral arteries, embryonic development, microanatomy, CT angiography, microdissection, corrosion casts

## Abstract

Fenestration of the intracranial artery is an anatomical remnant from the embryonic development of the vascular system. A cerebral aneurysm is a focal pathological dilation of the arterial wall. The occurrence of an aneurysm at the site of fenestration is rare in cerebral circulation but may have potential clinical implications. This study aimed to identify the frequencies of fenestrations and aneurysms, their locations, and their relationships. The vasculature of 35 adult brains was used for micromorphological dissection and analysis under a stereoscopic microscope, following an arterial injection with a mixture of formaldehyde, melted gelatin, and the solution of India ink. Additionally, we analyzed another group of vascular casts obtained from 15 brains injected with methyl methacrylate (MMA). A fenestration of the M1 segment of the middle cerebral artery (MCA) was sectioned for histological analysis. We also examined computed tomography (CT) angiograms of 1,230 patients, analyzed the data, and compared the findings with anatomical observations. In our group of 50 anatomical specimens, fenestrations were found in 12 brains (24%), affecting different cerebral arteries, with three cases showing double fenestrations on the same vessel. Aneurysms were observed in six brains (12%), always one per brain, with one case (2.00%) involving an aneurysm associated with the wall of a fenestration. Analysis of CT angiograms from 1,230 patients showed 26 arterial fenestrations (2.11%) in 26 patients, 28 aneurysms (2.28%), and one case (0.08%) where an aneurysm arose from a fenestration. The presence of an aneurysm on a fenestrated cerebral artery is a rare phenomenon, occurring far less frequently than isolated fenestrations or aneurysm formation.

## Introduction

The embryonic development of the vascular system follows the formation of the main arteries as channels passing through a capillary network (Padget, 1948). During the fusion of plexiform vessels, a small part of this network may persist, resulting in the formation of two arterial channels for a shorter or longer segment of their course. This variant of cerebral circulation, known as fenestration, appears as a localized dilation of the vessel with a central perforation, from which side branches arise from both channels (Moffat, 1962). Fenestration of a brain artery is characterized as a segmental, endothelium-lined duplication of the vessel with a central slit-like opening or as a window-like hole through the wall of an individual vessel in the brain (Menshawi et al., 2015; Van Rooij et al., 2015; Wu et al., 2020; Bertulli and Robert, 2021).

The embryonic development of the normal cerebral arterial system may influence the formation of cerebral aneurysms, which are focal pathological fusiform or saccular dilations of a weakened arterial wall resulting from local inflammation and degeneration. The association between fenestration and aneurysmal dilation is a rarely reported phenomenon, primarily found in anatomical descriptions or angiographic studies of cerebral arteries. A possible effect of locally altered blood flow at the site of fenestration may contribute to aneurysm enlargement (Chalouhi et al., 2013; Menshawi et al., 2015; Van Rooij et al., 2015; Wu et al., 2020; Bertulli and Robert, 2021; Kim et al., 2022).

### Brief basic cerebral vascular embryology

In the earliest stages of embryonic development, approximately 22 days into gestation, two longitudinal dorsal aortae (DA) give rise to a series of dorsal intersegmental arteries for each somite. The primitive internal carotid artery (ICA) appears in an embryo measuring 3 mm in length (approximately the 24th day of gestation) as a proximal continuation of each dorsal aorta. At approximately 4 mm in length during embryonic development (the 28th day of intrauterine life), the DA begin to fuse, forming the aorta (Padget, 1948). The dorsal cranial intersegmental arteries from the primitive ICA develop into two longitudinal neural arterial plexuses (LNAP), which later merge into a single basilar artery (BA) (Vasović et al., 2013; Bertulli and Robert, 2021). The LNAP receives the cranial continuation of the primitive ICA, supported by four transient connections between the primordial ICA and BA, known as carotid-basilar anastomoses: the primitive trigeminal, otic, hypoglossal, and proatlantal or first cervical intersegmental artery (Vasović et al., 2009; Wang and Yu, 2022). Simultaneously, the primitive ICA bifurcates at the level of the trigeminal ganglion and optic vesicle into two plexiform branches: the cranial and the caudal (Padget, 1948; Moffat, 1962).

The cranial branch of the ICA gives rise to the anterior choroidal artery, the middle cerebral arterial (MCA) plexus, and the primitive olfactory artery, the median part of which represents the future anterior cerebral artery (ACA). These are interconnected by a vascular plexus that will develop into the future anterior communicating artery. The caudal trunk of the ICA gives rise to the posterior choroidal, diencephalic, and mesencephalic arteries terminates as the posterior communicating artery, which soon joins the distal part of the basilar artery, forming the proximal P1 segment of the PCA (Padget, 1948; Vitošević et al., 2019).

At the 4 mm stage of embryonic development, a large lateral longitudinal artery (LLA) forms on the ventrolateral wall of the metencephalon. This bilateral arterial network originates from the dorsal cranial intersegmental arteries. The LLA is supplied cranially by lateral branches of the basilar artery and caudally by the first cervical intersegmental artery, also known as the proatlantal artery of Padget. It is a temporary vessel, often developing into an accessory anastomosis between the basilar and vertebral arteries, referred to as the primitive lateral basivertebral anastomosis. The longitudinal remnants of this lateral channel are often connected to the cerebral part of the primitive vertebral artery (VA) through transverse branches, maintaining a plexiform appearance in the arteries of the embryonic medulla (Padget, 1948; Lang, 1990; De Caro et al., 1995; Bertulli and Robert, 2021). At approximately 10 mm in length (33 days of development), the VA forms through the plexiform union of the upper six cervical intersegmental arteries, including the proatlantal artery (Moffat, 1962; Tetiker et al., 2014; Menshawi et al., 2015).

### Hemodynamics of cerebral aneurysms and arterial stenosis

The influence of hemodynamic forces is one of the factors in the development and progression of diseases affecting cerebral blood vessels. Predilection sites for the initiation and formation of aneurysms include points of bifurcation, branching, and the outer walls of curved arterial sections. The wall shear stress (WSS) at the impingement point caused by pulsatile flow may lead to structural endothelial damage. Aneurysm growth results from the consequential degeneration of the arterial wall due to endothelial inflammation and apoptosis of the smooth muscle layer. Aneurysm rupture occurs under the influence of hemodynamic forces: high WSS and elevated blood pressure. Computational fluid dynamics (CFD) analysis processes angiographic images into 3D geometric data. This approach creates an individual 3D model of blood vessels that simulates blood flow and provides better insight into aneurysm development and rupture, which is essential for planning aneurysm treatment (Jeong and Rhee, 2012).

Analyzing the hemodynamics of flow is essential for understanding the dramatic rupture of an aneurysm. This can be accomplished using CFD simulation to study the geometry of the aneurysm and the associated wall parameters: wall pressure and WSS, which increase with recirculation flow near the flow-wall interaction area inside the bulge (Rahma and Abdelhamid, 2023). A group of researchers examined the flow characteristics within the aneurysm model while varying flow rates. By using a CFD simulation, they analyzed hemodynamic parameters as the flow rate increased, leading to the visibility of the vortex and revealing extremely high WSS values in the dome area of the aneurysm and in the bifurcation zones (Souza et al., 2024).

Narrowing of the cerebral artery, known as stenosis, results in decreased blood flow through the vessel and a dangerous reduction in the brain’s blood supply. The significance of blood flow has led to the development of various mathematical models to depict hemodynamic phenomena. Advances in computer technology have facilitated simulations of blood flow under different conditions using CFD, including changes in arterial structure, such as stenosed arteries. Arterial diseases greatly affect hemodynamics due to alterations in WSS. A study utilizing CFD models demonstrated that blood flow varies with the degree of occlusion. A 90% occlusion is particularly clinically significant, as it increases WSS and establishes laminar flow, potentially leading to the rupture of atherosclerotic plaques from the artery wall and the formation of thrombosis (Roy et al., 2017). Clinical practice requires processing large amounts of data obtained from medical imaging techniques to predict blood hemodynamics in patients with arterial stenosis. The application of CFD mathematical techniques, a new prognostic method, allows for the simulation of blood flow rates and brain blood distribution following the specified procedure (Polanczyk et al., 2018). Similarly, the geometry of the human fenestration model should be utilized to simulate the hemodynamics of blood flow through the main arterial tree, which divides into two branches before merging again into a single artery.

This study aimed to identify the frequencies of fenestrations and aneurysms, their locations, and their relationships in two groups of specimens: anatomical and clinical. Understanding morphological variants and anomalies could provide the anatomical basis of embryonic and clinical significance for neurologists, neuroradiologists, and neurosurgeons.

## Materials and methods

This report focuses exclusively on comparing the frequencies of fenestrations and aneurysms in two groups of cases, making factors such as age, gender, and disease irrelevant to determining the origin of the sample. The metric characteristics of fenestrations and aneurysms are not included in this brief research report.

### Microanatomical dissection of injected cerebral arteries

A total of 35 adult human brains from the collection of the Laboratory for Vascular Morphology were used for micromorphological dissection and analysis. After ligating the damaged vessels, plastic catheters were inserted into both the internal carotid and vertebral arteries. The carotid and vertebrobasilar arterial systems were then flushed with an isotonic saline solution. A mixture of 4% buffered formaldehyde, melted gelatin, and a 10% solution of India ink was injected into the arterial vessels. After 4 weeks, the arteries of the fixed brains were carefully microdissected using microsurgical instruments, and the fibrous trabeculae of the arachnoid mater were carefully removed. The arterial branches were examined under a stereo microscope (Leica MZ6), accurate drawings of each specimen were created, and digital photos were taken. One terminal segment of the ICA, which bifurcates into the ACA and MCA and shows the fenestration of its M1 segment, was sectioned longitudinally and serially into 5 μm thick slices to depict the histological structure of the arterial wall. These slices were stained with hematoxylin and eosin (H&E), and the Masson trichrome methods were used.

### Microanatomical examination of vascular corrosion casts

We analyzed a group of corrosion vascular casts from the brain arteries obtained from 15 brains. A solution of methyl methacrylate, composed of monomer and polymer components with a specific color pigment and mixed shortly before use, was injected simultaneously into the carotid and vertebrobasilar arterial systems. After 5 h of polymerization and hardening, we applied a 40% solution of potassium hydroxide, necessary for the corrosion of soft tissue, over the following week. Once the brain tissue completely dissolved, the vascular plastic cast was washed in hot running water and dried. The corrosion-cast specimens of brain arteries were examined under a zoom microscope (Leica MZ6) and photographed with a digital camera (Leica DFC295). Using specific software (Leica Interactive Measurements), we analyzed various measurements regarding the length and diameter of the cerebral vessels in both groups of specimens. The study protocol was approved by the Ethics Committee of the Faculty of Medicine (No. 29/VI-1; Date 19-6-2013).

### CT angiography of brain vessels

This study analyzed the CT angiography results of cerebral vessels from 1,230 patients at our institution between January and December 2015. The morphological characteristics of the brain’s blood vessels, including fenestrations and aneurysms, were retrospectively examined using images obtained from a 128-multidetector scanner (Somatom Definition AS; Siemens Healthcare, Erlangen, Germany). During the procedure, an intravenous catheter was used to inject non-ionic iodinated contrast media (Ultravist 370, Bayer HealthCare Pharmaceuticals, Berlin, Germany) using an automatic injector at a rate of 4.5 mL/s (Medrad^®^ Stellant^®^ Dual Syringe CT Injection System, Indianola, PA, United States). The obtained images were transferred to a Syngo workstation for analysis. In addition to the axial source data, post-processed multiplanar reformatted (MPR), maximum-intensity projection (MIP), and 3D volume-rendering (VR) images were evaluated by an experienced team that included a neuroradiologist and a radiology resident, with decisions made by consensus. This protocol was approved by the authorities at the Clinic of Neurosurgery, the Department of Radiology, and the Ethics Committee of the University Clinical Centre.

### Statistical analysis

Statistically, the frequencies of fenestrations and aneurysms, as well as their parent arteries and metric characteristics, were examined. Quantitative experimental data were analyzed using IBM SPSS Statistics version 25.0 software (SPSS, Inc., Chicago, IL, United States). The statistical analyses included chi-square tests and Fisher’s exact tests to compare categorical variables. A probability level of *p* < 0.05 was considered an appropriate indicator of a statistically significant difference.

## Results

**Figure 1:**
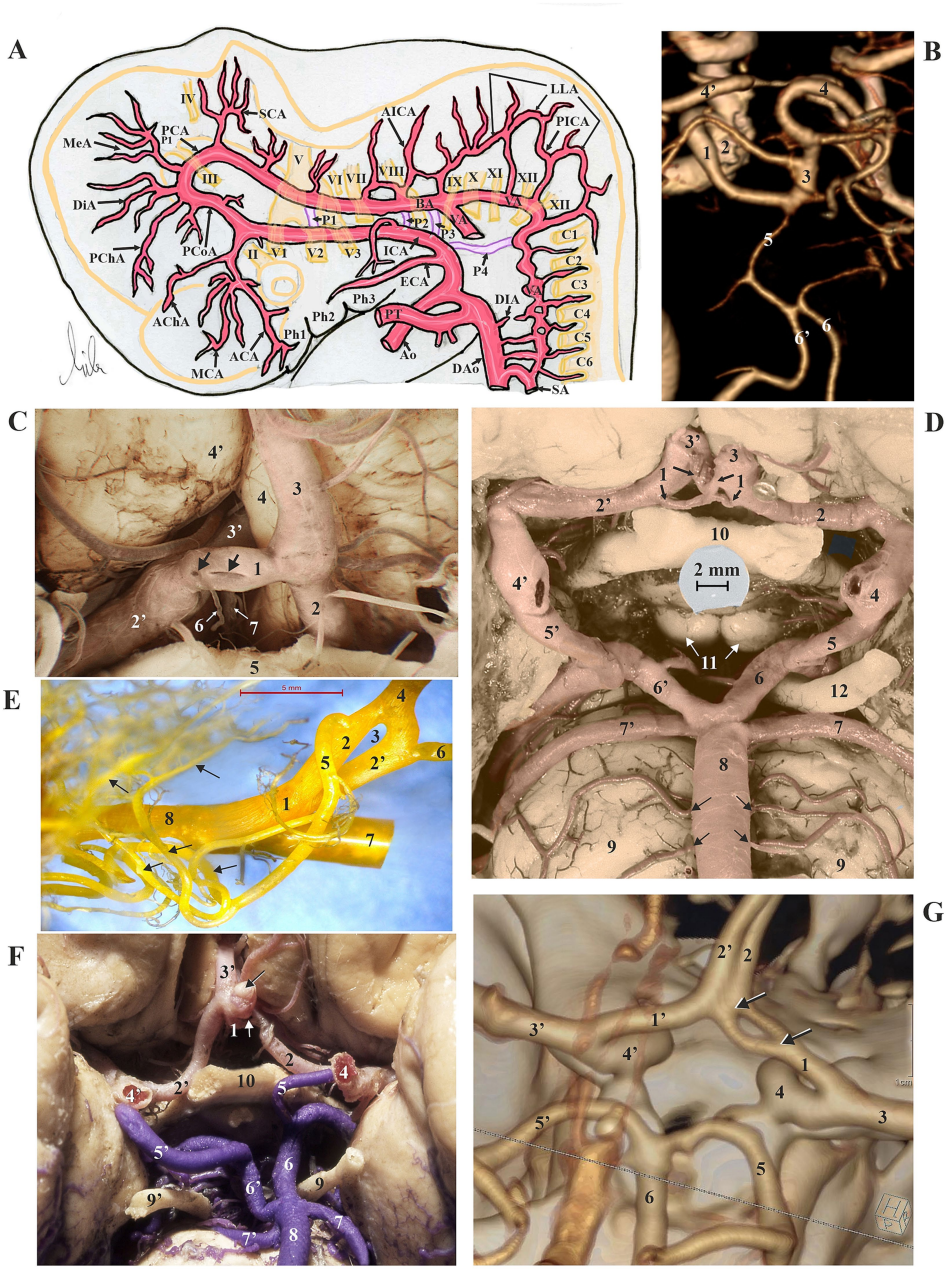
**(A)** The arterial system of a 36-day-old embryo at the 14 mm stage was modified after Padget (1948). For explanations of terms, refer to the list of abbreviations. **(B)** Dorsal view of the left persistent primitive trigeminal artery (1), extending from the left cavernous segment of the ICA (2) to the distal part of the BA (3); 4 and 4′, right and left P2 segments of the PCA; 5, hypoplastic proximal part of the BA; 6 and 6′, right and left hypoplastic VA. CT angiogram. **(C)** Ventral view of the anterior communicating artery, ACoA, (1) with pinhead-like and slit-like fenestrations (arrows). 2, left A1 segment of the anterior cerebral artery (ACA); 2′, right A1 segment of the ACA; 3, left A2 segment of the ACA; 3′, right A2 segment of the ACA; 4, left gyrus rectus; 4′, right gyrus rectus; 5, optic chiasm; 6, hypothalamic branch of ACoA; 7, subcallosal branch of ACoA. Ventral view of the central part of the brain; arteries were injected. **(D)** Plexiform ACoA (1) made of two interconnected channels with two fenestrations. 2, left A1 segment of the anterior cerebral artery (ACA); 2′, right A1 segment of the ACA; 3, left A2 segment of the ACA; 3′, right A2 segment of the ACA; 4, left internal carotid artery (ICA); 4′, right ICA; 5, left posterior communicating artery (PCoA); 5′, right PCoA; 6 and 6′, left and right P1 segments of the posterior cerebral arteries (PCA); 7 and 7′ left and right superior cerebellar arteries (SCA); 8, basilar artery (BA) with pontine branches (arrows); 9, pons; 10, optic chiasm; 11, mammillary bodies; 12, left oculomotor nerve. Ventral view of the central part of the brain; arteries were injected. **(E)** Case with saccular aneurysm (black arrow) on the right end of the ACoA (1) with a central fenestration (white arrow). 2, left A1 segment of the ACA; 2′, right A1 segment of the ACA; 3′, right A2 segment of the ACA; 4 and 4′, left and right ICA; 5 and 5′, left and right PCoA; 6 and 6′, left and right P1 segments of the (PCA); 7 and 7′, left and right SCA; 8, BA; 9 and 9′, left and right oculomotor nerves; 10, optic chiasm. Ventral view of the central part of the brain; arteries were injected. **(F)** Dorsal view of the left A1 segment of the ACA (1) showing two channels (2 and 2′) that form a fenestration (3) and unite in the left A2 segment of the ACA (4). The left Heubner’s artery (5), which sends numerous intracerebral branches (arrows), arises from the lateral component of the fenestration, while the ACoA (6) originates from its medial component. 7, left ICA; 8, left middle cerebral artery (MCA). Vascular corrosion cast. **(G)** Dorsal view of the right A1 segment of the ACA (1) exhibiting a fenestration (between the two arrows). 1′, the left A1 segment of the ACA; 2 and 2′, right and left A2 segments of the ACA; 3 and 3′, right and left middle cerebral arteries (MCA); 4 and 4′, right and left ICA; 5 and 5′, right and left P2 segments of the PCA; 6, BA. CT angiogram.

In the introductory section, this study provides a brief overview of embryonic brain artery development. A diagram of the arterial system of a 36-day-old embryo at the 14 mm stage (modified from Padget, 1948) is shown (Figure 1A), along with a CT angiogram illustrating a persistent primitive trigeminal artery, an early dorsal cranial intersegmental artery, and a carotid-basilar anastomosis, which is observed in one case (0.08%) in this study (Figure 1B).

### Vascular corrosion casts and dissected cerebral arteries

**Figure 2:**
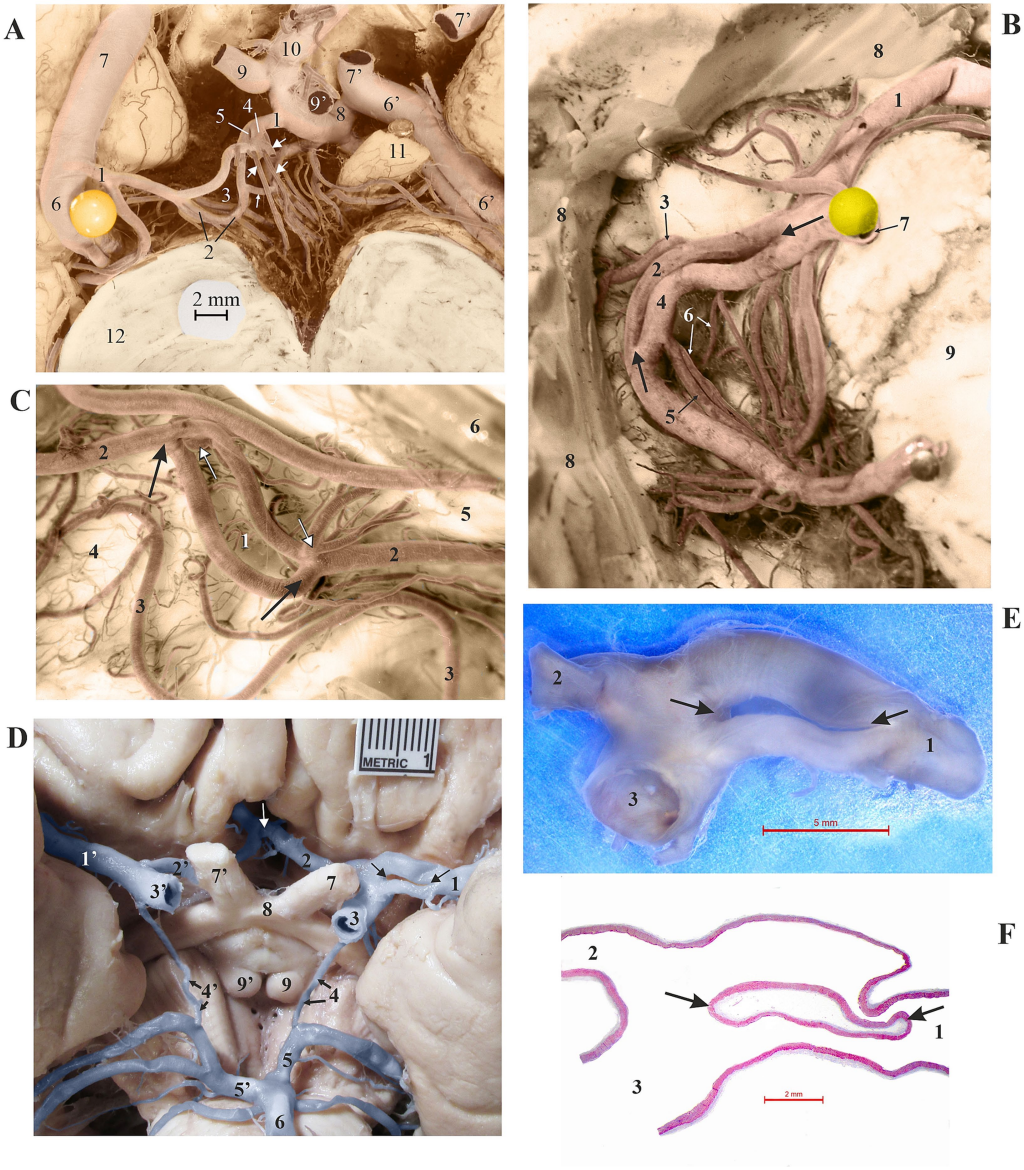
**(A)** Two fenestrations of the right hypoplastic P1 segment (1) of the PCA. One component (2) of the larger fenestration (3) sends two interpeduncular perforating arteries (cut) and an anastomotic vessel to the nearby perforating artery (small arrow). One channel (4) of the smaller fenestration (5) gives rise to four perforating arteries (large arrows). 6 and 6′, right and left P2 segments of the PCA; 7 and 7′, right and left (cut) PCoA; 8, left P1 segment of the PCA; 9 and 9′, right and left SCA (cut); 10, BA (elevated); 11, left oculomotor nerve; 12, mesencephalon. Ventral view of the central part of the brain; arteries were injected. **(B)** Fenestration (between two arrows) of the right P2 segment (1) of the PCA. The smaller component (2) of the fenestration gives rise to the lateral posterior choroidal artery (LPChA) (3). The larger component (4) provides the medial posterior choroidal artery (MPChA) (5) and two thalamogeniculate arteries (6). 7, common temporal artery (cut and removed); 8, temporal lobe (partially removed); 9, mesencephalon. Ventral view of the right part of the brain; arteries were injected. **(C)** Fenestration (1, between two black arrows) of the right MPChA (2) leads to two large thalamogeniculate arteries (white arrows). 3, right collicular artery; 4, right superior colliculus; 5, right medial geniculate body; 6, right pulvinar. Lateral view of the right side of the mesencephalon; arteries were injected. **(D)** Fenestration (between two arrows) of the left M1 segment (1) of the MCA. 1′, the right M1 segment of the MCA; 2 and 2′, left and right A1 segments of the ACA; 3 and 3′, left and right ICA; 4 and 4′, left and right PCoA; 5 and 5′, left and right P1 segments of the PCA; 6, BA; 7 and 7′, left and right optic nerves; 8, optic chiasm; 9 and 9′, mammillary bodies. Ventral view of the central part of the brain. **(E)** Cut and removed fenestration (between two arrows) of the left M1 segment (1) of the MCA from the previous case. 2, left A1 segment of the ACA; 3, left ICA. **(F)** Histological specimen of the same fenestration (between two arrows) of the left M1 segment (1) of the MCA from the previous image, stained using the Masson trichrome method. 2, left A1 segment of the ACA; 3, left ICA.

**Figure 3:**
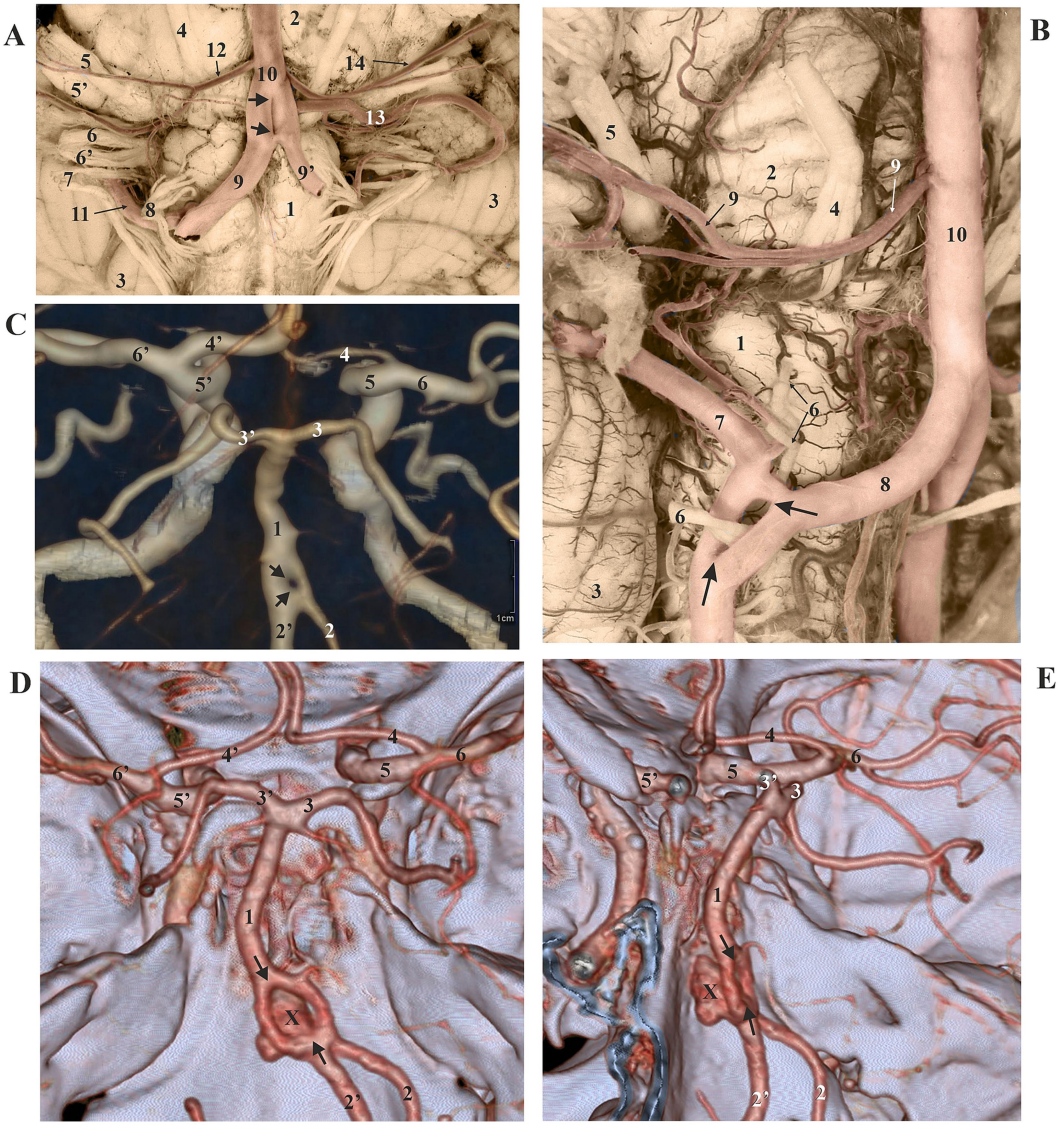
**(A)** Ventral view of the medulla (1), pons (2), and cerebellum (3), displaying the roots of the right abducens nerve (4), facial nerve, and vestibulocochlear nerves (5 and 5′); glossopharyngeal and vagus nerves (6 and 6′); accessory nerve (7); and hypoglossal nerve (8). The right and left vertebral arteries, VA (9 and 9′), partially merge in a slit-like fenestration (arrows) to form the BA (10). 11, right posterior inferior cerebellar artery (PICA) passing between the superior and inferior roots of the hypoglossal nerve (8); 12, right anterior inferior cerebellar artery (AICA); 13, left PICA; 14, left AICA. The arteries were injected. **(B)** Right ventrolateral view of the medulla (1), pons (2), and cerebellum (3), showing the roots of the right abducens nerve (4) (pierced by the pontine vein), the right facial nerve, the vestibulocochlear nerves (5), and the rootlets of the right hypoglossal nerve (6). The right PICA (7) arises from the lateral component of the fenestration (arrows) of the right VA (8), which contains the inferior roots of the hypoglossal nerve. 9, right AICA; 10, BA. Arteries were injected. **(C)** Dorsal view of the BA (1), featuring a fenestration (between two arrows). 2 and 2′, right (hypoplastic) and left VA; 3 and 3′, right and left P1 segments of the PCA; 4 and 4′, right (hypoplastic) and left A1 segments of the ACA; 5 and 5′, right and left ICA; 6 and 6′, right and left M1 segments of the MCA. CT angiogram. **(D)** Dorsal view of the BA (1), showing a significant fenestration (between two arrows) and a saccular aneurysm (X) originating from its proximal segment. 2 and 2′, right and left VA; 3 and 3′, right and left P1 segments of the PCA; 4 and 4′, right and left A1 segments of the ACA; 5 and 5′, right and left ICA; 6 and 6′, right and left M1 segments of the MCA. CT angiogram. **(E)** Left lateral view of the BA (1), displaying a large fenestration (between two arrows) and a saccular aneurysm (X) arising from its proximal segment, extending anteriorly and upwards. 2 and 2′, right and left VA; 3 and 3′, right and left P1 segments of the PCA; 4, right A1 segments of the ACA; 5 and 5′, right and left ICA; 6, right M1 segments of the MCA. CT angiogram.

Dissection of cerebral arteries in 35 injected brains and analysis of 15 corrosion casts revealed 12 arterial fenestrations (24%) across the 50 studied brains. In nine cases (18%), one fenestration was present per specimen, while in 3 brains (6%), double fenestration was found per case. The morphology of the anterior communicating artery (ACoA) can appear complicated and be formed by interconnected channels. ACoA fenestration was present in 3 (25%) brains, with two cases exhibiting double fenestration (Figures 1C,D,F). Fenestration of the ACA consistently appeared single in two (16.67%) of the explored brains with fenestrations. In one case, only the A1 segment of the ACA displayed the fenestration, whereas in the second case, both the A1 and A2 segments were involved in the formation of the fenestration (Figure 1E). Fenestration of the posterior cerebral artery (PCA) was infrequent, occurring in three of the cases (25%). One double fenestration was associated with the plexiform P1 segment, comprised of two independent openings, one smaller and the other larger (Figure 2A). The second fenestration in the P2 segment was a genuine duplication of a long segment of the PCA (Figure 2B). The third fenestration was located in the subthalamic part of the posterior medial choroidal artery (PMChA) (Figure 2C). Fenestrations and other variations of the middle cerebral artery (MCA) were also uncommon and less frequent than those in other cerebral arteries. The frequency of MCA fenestration was 8.33%, observed in only one brain (Figures 2D–F). Fenestrations of the ICA were absent in the examined brains. Basilar artery (BA) fenestrations were present in two brains (16.67%), always located in the proximal part, immediately after the union of the two vertebral arteries (Figure 3A). A rare fenestration of the VA, representing persistence of the caudal part of the primitive lateral basivertebral anastomosis, was identified in only one case (8.33%), where the inferior root fibers of the hypoglossal nerve passed through this arterial ring. In that case, the PICA arose from the lateral component of the fenestration of the parent VA (Figure 3B). The locations and frequency of the fenestrations are shown in Table 1.

**Table 1:** Locations and frequencies of cerebral fenestrations and aneurysms in two groups of specimens: anatomical and CT angiograms.

Cerebral artery fenestration and aneurysm		Location, number (%)							
		ACA	ACoA	MCA	ICA	PCA	BA	VA	Number total (%)
Fenestrations	**Anat. spec.**	2	3	1		3	2	1	12
	12 brains	(16.67)	(25.0)	(8.33)		(25.0)	(16.67)	(8.33)	
	50 brains	(4.0)	(6.0)	(2.0)		(6.0)	(4.0)	(2.0)	(24)
	**CTA**	6	5	1		1	12	1	26
	26 cases	(23.08)	(19.23)	(3.85)		(3.85)	(46.15)	(3.85)	
	1,230 cases	(0.49)	(0.41)	(0.08)		(0.08)	(0.98)	(0.08)	(2.11)
Aneurysms	**Anat. spec.**		1	2			3		6
	6 brains		(16.67)	(33.3)			(50)		
	50 brains		(2.0)	(4.0)			(6.0)		(12.0)
	**CTA**		5	7	12		4		28
	28 cases		(17.86)	(25)	(42.86)		(14.28)		
	1,230 cases		(0.41)	(0.57)	(0.98)		(0.33)		(2.28)
Fenestration an aneurysm	**Anat. spec.**		1						1
	12 brain		(8.33)						(8.33)
	50 brains		(2.0)						(2.0)
	**CTA**		2				1		3
	28 cases		(7.14)				(3.57)		(10.71)
	1,230 cases		(0.16)				(0.08)		(0.24)
Fenestration with aneurysm	**Anat. spec**.		1						1
	12 brains		(8.33)						(8.33)
	50 brains		(2.0)						(2.0)
	**CTA**						1		1
	28 cases						(3.57)		(3.57)
	1,230 cases						(0.08)		(0.08)

ACA, anterior cerebral artery; ACoA, anterior communicating artery; MCA, middle cerebral artery; ICA, internal carotid artery; PCA, posterior cerebral artery; BA, basilar artery; VA, vertebral artery.

The frequency of cerebral aneurysms in a group of 50 injected brains was 6 out of 50 (12%). Aneurysms most commonly arose from the basilar artery (BA) in three cases (50%), the MCA in two cases (33.33%), and the ACoA in one case (16.67%) (Figure 1F; Table 1). Only one case (8.33%) out of 12 showed the presence of cerebral artery fenestration along with an aneurysm in the same dissected specimen; this aneurysm was associated with the corner of the ACoA fenestration (Figure 1F; Table 1).

The analysis of stained longitudinal sections through the arterial wall and fenestration of the MCA showed no weak areas in the layers at any point in the vessel (Figure 2F).

### CT angiograms of brain vessels

We examined CT angiograms of 1,230 patients and found 26 (2.11%) arterial fenestrations among 26 patients, with one fenestration per patient. The detected fenestrations most frequently occur in the basilar artery (BA) in 12 cases (46.15%) (Figures 3C–E), the ACA in six cases (23.08%) (Figure 1G), the ACoA in five cases (19.23%), the MCA in one case (3.85%), the PCA in one case (3.85%), and the VA in one angiogram (3.85%) (Table 1).

In our clinical material consisting of CT angiograms from 1,230 patients, we identified 28 (2.28%) cerebral aneurysms. The most common origin of these aneurysms was the cerebral segment of the ICA in 12 (42.86%) cases, the MCA in 7 (25%) patients, the ACoA in 5 (17.86%), and the BA in 4 (14.28%) cases (Table 1). The incidence of cerebral aneurysms among patients with arterial fenestrations was 10.71% (3 out of 28 cases with aneurysms). Specifically, the ACoA was involved in two (7.14%) patients, and the BA was involved in one case (3.57%). Aneurysms associated with fenestrations were found very rarely, occurring only once at a rate of 3.75% (1 out of 28 patients), which is 0.08% when compared to the entire group of 1,230 patients. In that patient, the aneurysm arose from the proximal point of a fenestration of the BA (Figures 3D,E; Table 1).

We found statistically significant differences in the number of fenestrations between the anatomical and CTA specimens (24% vs. 2.11%), χ^2^ = 79.895 (*p* = 0.0001).

We found statistically significant differences in the number of aneurysms between the anatomical and CTA specimens (12 vs. 2.28%), χ^2^ = 17.569 (*p* = 0.0001).

We found statistically significant differences in the number of fenestrations and aneurysms in the same specimen between the anatomical and CTA specimens (2.0% vs. 0.24%), χ^2^ = 4.756 (*p* = 0.029).

Our report showed no statistically significant differences in the number of fenestrations associated with aneurysms in the same specimen between the anatomical and CTA specimens (2.0% vs. 0.08%), according to Fisher’s exact test (*p* = 0.0766).

## Discussion

The present study of anatomical specimens from 35 brains and 15 corrosion casts showed 12 (24%) arterial fenestrations. In three (6%) of the brains, we observed double fenestration. Among the CT angiograms of 1,230 patients, we found 26 (2.11%) arterial fenestrations across 26 patients, with one fenestration per patient. This statistically significant difference in reported incidences of arterial fenestrations may be explained by the higher sensitivity of anatomical dissections of injected cerebral arteries observed under a stereoscopic microscope compared to CT angiographic reports (Cooke et al., 2014). Fenestrations in smaller arteries, such as the plexiform ACoA or the hypoplastic P1 segment of the PCA, are often not visible, and if one does not specifically search for fenestrations while interpreting the images, they may indeed be overlooked (Milisavljević et al., 1986). Anatomical studies focused on the fenestrations of all cerebral arteries are rare, and analyses of cases where they are associated with aneurysms are almost non-existent. An anatomical study involving 333 formalin-fixed brains found a very high incidence of fenestrations, with up to 41% of cases reported, most frequently found in 79% of the brains concerning ACoA (Krystkiewicz et al., 2021). In our view, only brain specimens with arteries injected with various fillers, such as India ink and methyl methacrylate, can provide an accurate representation, particularly in cases of very small fenestrations, especially when examining the ACoA. The side branches of the ACoA, such as the small hypothalamic or larger subcallosal arteries, along with numerous anastomoses between the interpeduncular branches of the P1 segment of the PCA, particularly in non-injected vessels, can create an appearance suggestive of possible fenestrations (Milisavljević et al., 1986).

Previous studies retrospectively reviewed 10,927 digital angiograms and identified fenestrations in 228 patients (2.1%) and 208 fenestrations (1.13%) in 18,360 patients, a percentage similar to that in our clinical study (Cooke et al., 2014; Wu et al., 2020). In another study using 3D rotational angiography, 140 patients were analyzed, confirming 33 (24%) fenestrations, matching the same percentage observed in our anatomical study (Van Rooij et al., 2015). Analyzing the distribution of fenestration locations, the same group of authors demonstrated that 87% occurred in the anterior circulation, primarily with the ACoA as a parent artery (69%), while only 13% were found in the posterior circulation (Van Rooij et al., 2015). In their study, Cooke et al. (2014) found a predominance of fenestrations within the posterior circulation (73.2%), most frequently affecting the basilar artery (52.6%). Our findings confirmed an equal distribution of fenestrations in the anterior and posterior parts of the cerebral circulation in both anatomical (50% versus 50%) and clinical (46.16% versus 53.84%) groups.

The two most exposed and affected cerebral arteries due to fenestrations are the ACoA and BA (Cooke et al., 2014; Van Rooij et al., 2015; Wu et al., 2020; Krystkiewicz et al., 2021; Xu et al., 2022). At the 20–24 mm (~45 days) stage 6 of Padget, the embryonic circle of Willis begins to form, with the ACoA exhibiting a plexiform structure (Bertulli and Robert, 2021). The adult ACoA exhibits a variable appearance; it can be plexiform or resemble the letters H, Y, X, or O, as opposed to a single channel-like vessel (Chen et al., 2020). ACoA fenestration has been observed in 11.25% (Xu et al., 2022), 21% (Chen et al., 2020), 38.5% (Cooke et al., 2014), and 69% of brains (Van Rooij et al., 2015), compared to our findings of 19.23% in CT angiograms and 25% from anatomical collections with fenestrations. The majority of fenestrations in the BA originate from its embryonic development (Padget, 1948). The BA develops from two LNAP, which subsequently fuse to form a single basilar artery (Bertulli and Robert, 2021). BA fenestration has been noted in 9% (Van Rooij et al., 2015), 35% (Xu et al., 2022), 50.24% (Wu et al., 2020), and 52.6% of brains (Cooke et al., 2014). Our study reveals BA fenestration in 46.15% of 26 cases identified through CT angiograms and in 16.67% of 12 brains found in anatomical collections (4% of 50 brains).

Cerebral aneurysms (CAs) are focal pathological dilatations of the arterial wall that result from inflammation and degeneration. CAs are found in 1–5% of the population (Chalouhi et al., 2013; Brown and Broderick, 2014). Endothelial changes and dysfunction in response to hemodynamic stress play a key role in the formation and development of CAs (Chalouhi et al., 2013). Even small CAs pose a risk of growth and rupture, and MRA or CTA, along with interventional treatment, is recommended (Yoon et al., 2004; Fujimoto et al., 2007; Im et al., 2007; Tanaka et al., 2012; Brown and Broderick, 2014; Filep et al., 2022). Fenestrations are important from a surgical perspective when unexpectedly present in the BA or MCA regions, as they help prevent artery rupture during mechanical thrombectomy (Zhang et al., 2022). The frequency of CAs in a sample of 50 injected brains was 6/50 (12%), with 1/12 (8.33%) in cases where fenestration was present in the same brain. In only one case (8.33%) out of the 12 fenestrations was the aneurysm associated with the end of the fenestration of the ACoA (2% of the 50 studied brains). The increased number of aneurysms in our anatomical specimens may be attributed to the age group (over 75 years) of individuals who participated in the body donation program for medical schools (Imaizumi et al., 2018).

In our clinical analysis of CT angiograms from 1,230 patients, we identified 28 (2.28%) aneurysms, with 3 out of 28 (10.71%) occurring in cases where fenestration existed in the same brain. In a large retrospective study involving 3,049 patients, the authors found 137 (5%) aneurysms following MRA examinations. Intracranial aneurysms were most commonly localized in the distal segment of the ICA, with 64 (39%) cases, and the MCA, with 40 (24%) cases (Jeon et al., 2011). The distribution of aneurysms in our CTA group is almost identical, at 42.86 and 25%, respectively, out of the total 2.28% identified. Another report from a second group of researchers analyzed 4,070 MRA scans of healthy individuals and revealed 176 (4.32%) aneurysms, primarily located in the ICA, with 148 (78.7%), followed by the ACoA with 22 (11.7%) and the MCA with 17 (9.00%) (Imaizumi et al., 2018). Our results of 2.28% aneurysms found in the CTA group also indicated a lower incidence of ICA cases, along with higher occurrences of ACoA and MCA aneurysms at 39, 17.86, and 25%, respectively.

The incidence of CAs associated with fenestrations in this patient group was found to be very rare, occurring in only one case, 3.57% (1 out of 28 patients with aneurysms), which equates to 0.08% of the entire cohort of 1,230 patients. When comparing our results—the frequency of CAs at the site of fenestration of 1 (0.08%) case in 1,230 patients, or in 1 (2%) case in 50 anatomical specimens—with the study by Wu et al. (2020), which reported only one aneurysm at the site of fenestration (out of 211 fenestrations, 0.47%), and another report documenting 10/208 (4.5%) CAs located on a fenestration (Van Rooij et al., 2009), along with the findings of four cases (1.2%) on anatomical material by Krystkiewicz et al. (2021), we cannot conclude that all these studies indicate a clear clinical association between aneurysms and fenestrations.

A potential limitation of our report is the relatively small anatomical sample, which consists of 35 brains with injected arteries and 15 corrosion casts of brain arteries. Both groups comprise human specimens that must be in stable condition without any preservation before injection. The collection, preparation, and analysis of these specimens are a long and meticulous process. However, the final results provide highly reliable representations of the real studied morphology.

## Conclusion

Fenestration is an embryonic variant of cerebral circulation occurring in both the anterior and posterior cerebral circulation. Findings from our studies on brain artery fenestrations associated with cerebral aneurysms indicate that fenestration can very rarely be associated with intracranial aneurysms, with a possible minor influence on aneurysm development. However, a change in blood flow at the site of fenestration may contribute to the enlargement of an existing aneurysm, similar to occurrences at other arterial bifurcations or branch points. Understanding the specific microanatomical characteristics of cerebral arteries, fenestrations, and aneurysms is of significant neuroradiological and neurosurgical importance.

## Data Availability

The raw data supporting the conclusions of this article will be made available by the authors, without undue reservation.

## Glossary

ACA: Anterior cerebral artery
AChA: Anterior choroidal artery
AICA: Anterior inferior cerebellar artery
Ao: Aorta
BA: Basilar artery
C1-C6: First to sixth cervical spinal nerves
**Cranial nerves**
II: Optic nerve
III: Oculomotor nerve
IV: Trochlear nerve
V: Trigeminal nerve
V1: Ophthalmic division
V2: Maxillary division
V3: Mandibular division
VI: Abducens nerve
VII: Facial nerve
VIII: Vestibulocochlear nerve
IX: Glossopharyngeal nerve
X: Vagus nerve
XI: Accessory nerve
XII: Hypoglossal nerve
DAo: Dorsal aorta
DiA: Diencephalic artery
DIA: Dorsal intersegmental artery
**Dorsal cranial intersegmental artery**
LNAP: Longitudinal neural arterial plexus
P1: Primitive trigeminal artery
P2: Primitive otic artery
P3: Primitive hypoglossal artery
P4: Proatlantal artery of Padget
ECA: External carotid artery
ICA: Internal carotid artery
LLA: Lateral longitudinal artery
LPChA: Lateral posterior choroidal artery
MCA: Middle cerebral artery
MeA: Mesencephalic artery
MPChA: Medial posterior choroidal artery
PCA: Posterior cerebral artery
PChA: Posterior choroidal artery
PCoA: Posterior communicating artery
Ph1-Ph3: First to third pharyngeal arches
PICA: Posterior inferior cerebellar artery
PT: Pulmonary trunk
SA: Subclavian artery
SCA: Superior cerebellar artery
